# The genetic and epigenetic contributions to the development of nutritional rickets

**DOI:** 10.3389/fendo.2022.1059034

**Published:** 2022-12-22

**Authors:** Innocent Ogunmwonyi, Adewale Adebajo, Jeremy Mark Wilkinson

**Affiliations:** Department of Oncology and Metabolism, University of Sheffield, Sheffield, United Kingdom

**Keywords:** epigenetic, genetics, bone metabolism, rickets, nutrition – clinical

## Abstract

Nutritional rickets is an important disease in global health. Although nutritional rickets commonly manifests as bony deformities, there is an increased risk of life-threatening seizures secondary to hypocalcaemia. Dietary vitamin D deficiency is associated with the development of nutritional rickets among children and infants. This is especially true in populations of darker skinned individuals in high-latitude environments due to decreased ultraviolet light exposure, and in populations in tropical and subtropical climates due to cultural practices. A growing body of evidence has demonstrated that genetic factors might influence the likelihood of developing nutritional rickets by influencing an individual’s susceptibility to develop deficiencies in vitamin D and/or calcium. This evidence has been drawn from a variety of different techniques ranging from traditional twin studies to next generation sequencing techniques. Additionally, the role of the epigenome in the development of rickets, although poorly understood, may be related to the effects of DNA methylation and non-coding RNAs on genes involved in bone metabolism. This review aims to provide an overview of the current evidence that investigates the genetic and epigenetic determinants of nutritional rickets.

## Introduction

Rickets is a disease of thegrowth plate cartilage which occurs primarily in infants and young children. This occurs due to inadequate mineralisation of the osteoid secondary to hypocalcaemia or hypophosphatemia, or through direct inhibition of mineralisation by minerals such as fluoride and aluminium (1, 2).

In growing long bones, osseous tissue is created from cartilage by endochondral ossification, where hypertrophic chondrocytes in the ossification centre produce extracellular matrix that is then mineralised in the presence of adequate calcium and phosphate. Calcified cartilage is subsequently reabsorbed and replaced by woven and finally lamellar bone (3). Initiation of the ossification process involves chondrocyte apoptosis that is mediated by phosphorylation of MAPK pathway intermediates by extracellular phosphate (4). Low serum calcium causes a compensatory hyperparathyroidism and subsequent hypophosphataemia, inhibiting the normal process of chondrocyte apoptosis (4, 5). This results in the accumulation of hypertrophic chondrocytes, eventually leading to abnormal growth of the cartilaginous epiphyseal plate and the deposition of osteoid under the growth plate (1, 2). This ultimately gives rise to the characteristic phenotypical changes and clinical signs associated with rickets , including bone deformities (such as bow-legs and knock-knees), developmental delay, and the increased risk of hypocalcaemic seizures, cardiomyopathy (in infants) and death (6). The diagnosis of rickets is commonly made *via* a combination of radiological, clinical, and biochemical criteria (as reviewed by Munns et al.) (7).

Rickets can be a consequence of several aetiologies, including mutations directly affecting genes involved in bone homeostasis, mineralisation and renal metabolism, acquired impairments in the renal tubular handling of minerals (due to drugs such as tetracyclines and conditions such as Fanconi’s anaemia) and acquired deficiencies in metabolism (such as liver disease) (1, 8, 9). These aetiologies, while important, are described in more detail in other review articles (1–3). Rickets secondary to nutritional deficiencies is the most common cause of rickets globally and is primarily caused by insufficient calcium, vitamin D and/or phosphate intake. The prevalence of nutritional rickets varies, with a relatively low incidence seem in high income countries such as the UK and Ireland, (at 0.48 cases per 100,000) and Canada (at 2.9 cases per 100,000) (10, 11). However, nutritional rickets appears to be more common in low- and middle-income countries in Asia and Africa. For example, there is an estimated 2.7 cases per 1000 children in India based on radiographs, and there is a reported prevalence of 3.3% in Gambian children based on clinical examination (12, 13). However, some inter-study variation can be attributed to differences in diagnostic methods (e.g., clinical vs radiological vs biochemical diagnoses) and the possible selection of subpopulations which may have a higher prevalence of rickets (14).

Calcium- deficiency rickets can be caused by a lack of dietary calcium (even though the fractional intestinal calcium absorption may be maximised) or insufficient intestinal calcium absorption due to either a lack of dietary vitamin D or a lack of its active metabolite, 1,25(OH)_2_D (15, 16). Calcium deficiency rickets secondary to vitamin D deficiency involves overlapping contributions from cultural, environmental, and genetic factors. For example, a lack of UVB exposure limits the production of vitamin D_3_. UVB exposure is inhibited by melanin, which is produced in higher concentrations in darker skinned individuals (17). Behavioural and cultural practices, such as living predominantly indoors have also been associated with the development of nutritional rickets due to decreased UVB exposure (15). Diets low in dairy and vegan diets may result in an inadequate vitamin D and/or calcium intake (15, 18). However, even within communities that exhibit similar behaviours there can be inter-individual variation in the susceptibility of developing NR, baseline 25(OH)D levels and variation in the response to vitamin D supplementation (19, 20). Advances in our understanding on how the genome and epigenome can influence the expression of different phenotypical traits has led to an increased understanding of why this might occur and will be discussed in this review.

## The metabolism of vitamin D [25(OH)D]

**Figure 1:**
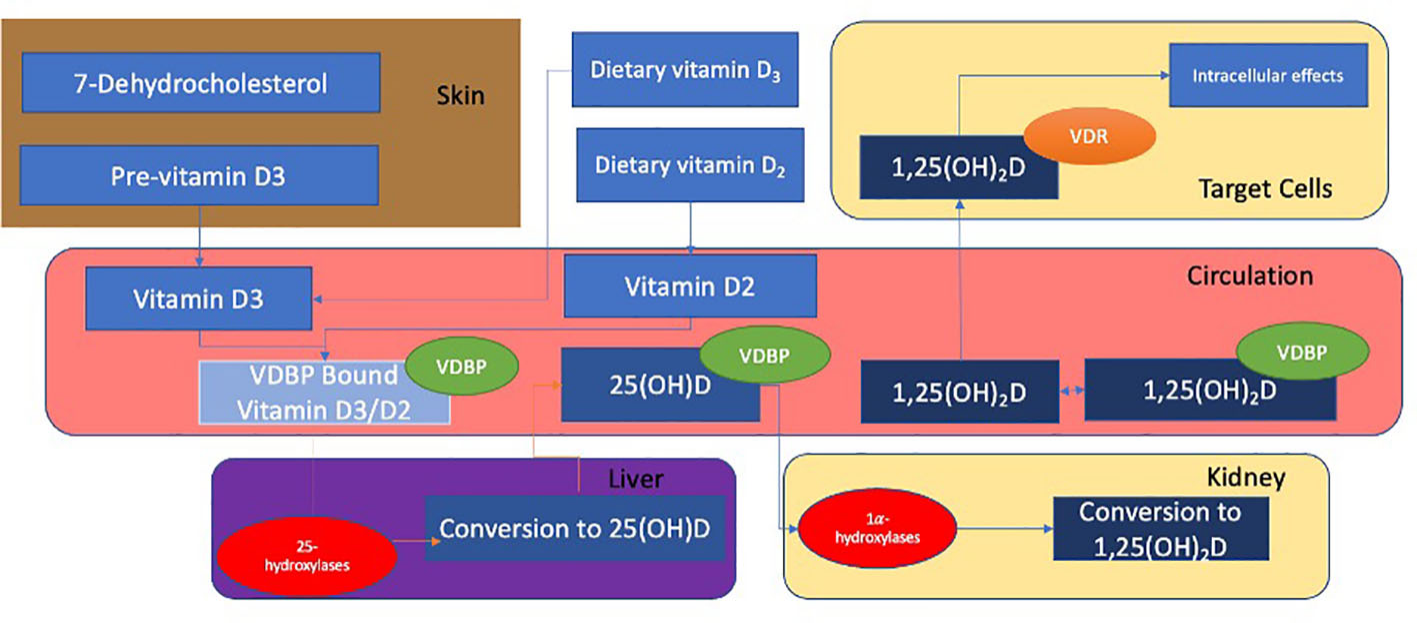
Vitamin D_2_ is synthesised from ergosterol found in yeast and fungi (17). Ultraviolet B (UVB) rays act on 7-dehydrocholesterol to form previtamin-D_3_ in the skin which subsequently undergoes a temperature-dependant isomerisation to vitamin D_3_. Vitamin D binding protein (VDBP) transports vitamins D_2_ and D_3_ to the liver where they undergo 25-hydroxylation to calcidiol (also known as 25(OH)D). This is mediated by 25 hydroxylases such as CYP2R1 (21). 25(OH)D is bound to VDBP and transported to the kidney, where it is filtered through the glomerulus and subsequently reabsorbed into the renal proximal tubular epithelium. This reabsorption occurs through endocytosis and is mediated by the cell surface receptors megalin and cubulin (22). 25(OH)D undergoes 1α-hydroxylation in the kidney and is converted to calcitriol (also known as *1*,*25*-(*OH*)2D), the active metabolite of 25(OH)D (22, 23). This is mediated by alpha hydroxylases, (such as CYP27B1). 25(OH)D and 1,25(OH)_2_D are inactivated by CYP24A1 in the kidneys (24).

Most cases of nutritional rickets arise from dysfunctional vitamin D metabolism. The normal metabolism of Vitamin D, as shown in **Figure 1**, results in the formation of the biologically active 1,25(OH)_2_D involving several steps in the skin, liver and kidneys (17, 21, 22). 1,25(OH)_2_D interacts with the intracellular vitamin D receptor (VDR), resulting in the formation of a VDR-retinoid receptor X (RXR) heterodimer. The VDR-RXR heterodimer binds to vitamin D response elements (VDREs) that act as co-activators or co-repressors of vitamin D responsive genes (25). Although the main physiological function of 1,25(OH)_2_D is the stimulation of intestinal absorption of calcium and phosphate (through upregulation of the TRPV6 channels), the VDR is expressed in a wide range of tissues, and It is estimated that approximately 3% of the human genome is regulated, either directly or indirectly, by the vitamin D endocrine system (5). Although the cellular action of vitamin D is primarily dependent on the production and delivery of 1,25(OH)_2_D, 25(OH)D is the most abundant circulating vitamin D metabolite and is more commonly used as an index of vitamin D status (17).

The synthesis and degradation of 1,25(OH)_2_D is regulated by parathyroid hormone (PTH), foetal growth factor 23 (FGF23) and 1,25(OH)_2_D itself, and mainly occurs at the 1-alpha hydroxylation step in the kidney (24). PTH stimulates renal CYP27B1 expression in response to hypocalcaemia and hypophosphataemia. Conversely, FGF23 is secreted by osteoblasts and osteocytes in response to circulating 1,25(OH)_2_D and downregulates the expression of CYP27B1. This is mediated by *α*KLOTHO (24). FGF23 also stimulates the production of CYP24A1, to increase the rate of degradation of 25(OH) and 1,25(OH)_2_D (26).

## The genetic contribution to nutritional rickets

Our current understanding of the underlying genetic contributions to nutritional rickets has developed through twin and family linkage approaches, as well as by candidate gene and genome wide association studies.

### Twin and family studies

Twin studies look at the extent to which an observed phenotypic variance of a trait can be explained by heritability. Using monozygotic (MZ, genetically identical) or dizygotic (DZ, non-identical) twins, the variation in susceptibility to a trait is split into three components known as the ‘ACE model’. The ‘A’ represents the sum of the ‘additive genetic components’ (which are the allelic effects that contribute to a phenotype), ‘C’ represents the shared environmental variance (such as shared parental habits and socioeconomic status in childhood), and ‘E’ represents individual environmental sources (such as unshared peers and individual events such as illness). The heritability of a trait can then be estimated by comparing MZ and DZ pairs using various theoretical models (27, 28). Although there are no twin studies that examine nutritional rickets directly, several twin and family studies have reported varying levels of heritability of 25(OH)D and 1,25(OH)_2_D (as summarised in **Table 1**).

**Table 1:** Twin and familial studies of circulating 25(OH)D and 1,25(OH)_2_D levels with measures of heritability (0.1 = 10%).

Sample size (MZ/DZ)	Type of study	Population	Country	Heritability estimate [25(OH)D]	Heritability estimate [1,25(OH)_2_D]	Reference
1068 (384/684)	Twins	Caucasian	UK	0.43 (0.28–0.57)	0.65 (0.53 – 0.74)	Hunter et al. (29)
947 (124 families)	Family	Caucasian Asthma patients	Germany	0.8	0.3	Wjst et al. (30)
198 (40/59)	Twins	Caucasian Multiple Sclerosis patients	Canada	0.77	–	Orton et al. (31)
513	Family	African American	USA	0.23 ± 0.11	0.20 ± 0.09	Engelman et al. (32)
513	Family	Hispanic American	USA (Hispanic, AA)	0.28 ± 0.10	0.16 ± 0.08	Engelman et al. (32)
504	Family	Hispanic American	USA (Hispanic)	0.41 ± 0.10	0.48 ± 0.10	Engelman et al. (32)
226 (951/58)	Twins	Asian	Philippines	0.69 (0.26 – 0.79)	–	Arguelles et al. (33)

MZ, monozygotic; DZ, dizygotic.

These studies give heritability estimates ranging from 16- 80% (29–33). The first twin study on vitamin D heritability by Hunter et al. reported the heritability of 25(OH)D and 1,25(OH)2D levels in 1068 European twin pairs (29) as 43% for 25(OH)D and 65% for 1,25(OH)_2_D. This has been corroborated by studies in families with co-morbidities including asthma and multiple sclerosis, as described in **Table 1**. The evidence from non-European cohorts, however, remains relatively sparse. Engelman et al’s family studies using Hispanic and African American populations reported an estimated heritability of 23 – 41% of 25(OH) and 20-48% of 1,25(OH)_2_D (32). Additionally, Arguelles et al’s twin study of vitamin D heritability in an Asian population of 109 Filipino twin pairs reported a heritability of 69% for 25(OH)D, and suggested that approximately half of the total additive genetic variation in 25(OH)D could be accounted for by genetic variation in skin colour as well as sun exposure behaviour (33).

Although the cumulative evidence from these studies suggests a substantial genetic component to serum 25(OH)D levels, this approach is limited in developing our understanding of the genetic component of nutritional rickets for several reasons. The first set of limitations are the general assumptions used in twin studies. The first is that MZ twins share 100% and DZ twins 50% of their genes, respectively. However, this assumption is not always true. Phenotypic discordance has been observed in MZ twins in traits such as birth weight and in the manifestation of several diseases which can be attributed to genotype alone, implying that less than 100% of genes may be shared (34). Several mechanisms have been proposed for MZ discordance, including post-zygotic mutation events, differences in DNA methylation patterns (due to differences within the intrauterine environment) and mosaicism due to placental stem cell transfer between twins (35). This assumption also relies on the absence of assortative mating, where mating is established based on phenotype (observable characteristics) (27). Ethnicity, culture, and ancestry are important determinants of social assortative mating, and DZ twins in populations demonstrating positive assortative mating practises may share more than 50% of their genes (36, 37). Additionally, there is the ‘equal environment’ assumption, which is that MZ and DZ pairs are exposed to shared environmental factors in similar degrees, whereas in reality MZ pairs may share more similar environments (37).

There are also limitations that are specific to the twin studies reviewed here. Firstly, the lack of non-European data means that heritability estimates may be different between populations. Discrepancies in the heritability estimates seen in **Table 1** could be attributed to confounding environmental variables or differences in study design, such as the methods of measurement of either 25(OH)D and 1,25(OH)_2_D heritability or allele frequencies between cohorts. Furthermore, current twin studies have focused on the heritability of 25(OH)D and 1,25(OH)_2_D levels. Although 25(OH)D/1,25(OH)_2_D levels are a commonly used surrogate marker for bone health indicating the risk of developing nutritional rickets, other factors that are independent of vitamin D and/or calcium status also affect the heritability of rickets. Unfortunately, there are no twin or family studies that have researched the heritability of nutritional rickets based on biochemical or clinical features. However, chronically low 25(OH)D levels are associated with the development of nutritional rickets, and by determining that there is a heritable component to 25(OH)D status, these early studies allow us to infer that there may be a genetic component to nutritional rickets. However, this would need to be supplemented by additional evidence, such as from well-powered twin and family studies that look at clinical or biochemical measures of nutritional rickets.

### Linkage studies

Genetic linkage refers to the observation that adjacent DNA sequences on a chromosome tend to remain linked during meiosis, and therefore segregate together significantly more often than if segregation occurred at random (38). By identifying markers that co-segregate with the disease of interest, genes associated with the disease can be tracked within a family. For the majority of the twentieth century, this was the primary genetic mapping tool for Mendelian traits and for complex traits with familial aggregation. This approach has been successful in mapping several forms of inherited rickets, including X-linked hypophosphataemic rickets and vitamin D- dependant rickets (38, 39). Linkage studies have been less successful in examining nutritional rickets, although Wjst et al. demonstrated that the DS2153 marker reached the threshold for genome wide significance (LOD = 3.4) (40). This is a region on chromosome 2p.16.2 that contains genes that are closely associated with vitamin D responsive elements, such as TGF*α*. However none of these genes have been implicated in the development of nutritional rickets, and there are no linkage studies that explore the diagnosis or severity of nutritional rickets (40). The relative difficulty in using linkage studies to investigate nutritional rickets is due to the variability in the severity of nutritional rickets as well as the traits involved in its development, such as vitamin D or calcium metabolism.

## Candidate gene studies

Other early studies looking at the genetic contribution to nutritional rickets have used a candidate gene approach, in which genes are selected based on *a priori* knowledge of their biological function. Here, single nucleotide polymorphisms (SNPs) are observed for their correlated occurrence against a particular trait across unrelated individuals. Several genes underlying the pathophysiological process of nutritional rickets have been studied and SNPs implicated in nutritional rickets are summarised in **Table 2** (41–46).

**Table 2:** Table of SNPs associated with nutritional rickets.

Gene	Gene position		SNP	SNP Position	SNPlocation	Study Population	Sample size	Investigation	Findings		Author
*GC*	4q12-q13		rs4588	72618323	Exon	Han Chinese children	506	Risk of NR vs wild type allele (Clinical and Radiological Diagnosis)	Increased risk of NR		Zhang et al. (41)
									*P* = 0.003, OR: 0.583, 95% CI: 0.412-0.836		
			rs222020	72618334	Exon				Increased risk of NR *P* = 0.009, OR: 1.526, 95% CI: 1.117-2.0985;		Zhang et al. (41)
			rs2282679	72608383	Intron				Increased risk of NR *P* = 0.010, OR: 0.636, 95% CI: 0.449-0.900		Zhang et al. (41)
			rs2298849	72648851	Intron				*P* = 0.001, OR: 1.709, 95% CI: 1.250-2.338		Zhang et al. (41)
*VDR*	12q13.11		rs2228570 Fok1	48272895	5′UTR	Egyptian and Turkish children	148	Prevalence of NR (Clinical and Radiological Diagnosis)	F allele frequency increased in NR patients (0.75 *vs.* 0.6, P = 0.024) f allele frequency decreased in NR patients (0.25 *vs.* 0.35, *P* = 0.024)		Baroncelli et al. (42)
						Nigerian children	199	Allele frequency and prevalence of NR (Clinical and Radiological diagnosis)	f allele frequency decreased in NR (0.17 vs 0.26, P = 0.03) F allele frequency increased in NR		Fischer et al. (43)
						Egyptian children	109	Clinical severity on X ray (Clinical and radiological diagnosis)	Ff genotype associated with lower X ray scores (−0.59±0.16 (P< 0.0006))		El Kholy et al. (44)
						Han Chinese children	159	Allele frequency in NR vs control groups (Unknown diagnosis)	f allele frequency decreased in NR patients (0.37 vs 0.54, P <0.01) F allele frequency increased in NR group (0.63 v 0.46, P <0.01)		Wu et al. (45)
			rs1544410 Bsm1	47846052	3’UTR	Egyptian and Turkish children	148	Severity of rickets (X-ray score) (Clinical and Radiological Diagnosis)	BB genotype associated with higher X-ray scores (P <0.05)		Baroncelli et al. (42)
			RS7975232 Apa1/VDR-24	47845054	Intron	Turkish children	24	Allele frequency in NR vs control groups (Biochemical and Radiological Diagnosis)	A allele frequency increased in NR patients (0.83 vs 0.57, P <0.01)		Bora et al. (46)
								Time to radiologic healing with treatment (Clinical and Radiological Diagnosis)	Minor allele homozygous (aa) associated with shortest time to healing (−0.32±0.12 (0.0107))		El Kholy et al. (44)
			Time to ALP normalisation (Clinical and Radiological Diagnosis)	Minor allele homozygous (AA) associated with shorter time to ALP normalisation (−0.52±0.21 (0.0165))		El Kholy et al (44)
			rs7305032 VDR 18	47856077	Intron	Egyptian children	109	Allele frequency in NR patients (Clinical and Radiological Diagnosis)	Major allele (TT) genotype frequency increased in NR patients with adequate sun exposure and inadequate calcium intake (P=0.02)		El Kholy et al. (44)
						Egyptian children	109	X-ray severity score (Clinical and Radiological Diagnosis)	Minor allele (CC) frequency associated with lower x-ray score (−1.05±0.4 (P =0.0109)		El Kholy et al. (44)
rs2525044 VDR-21	47848473	Intron	Egyptian children	109	Allele frequency in NR patients (Clinical and Radiological Diagnosis)	Major allele (CC) genotype frequency increased in NR patients with adequate sun exposure and inadequate calcium intake (P =0.03)		El Kholy et al (44)
rs10875695 VDR 7	47899254	Promoter	Egyptian children	109	Time to ALP normalization (Clinical and Radiological Diagnosis)	Allele genotype linked with reduced time to ALP normalisation (−0.42±0.13 (0.0020)		El Kholy et al (44)
*CYP2PR* *1*		11p15.2	rs10741657	14914878	5′ UTR	Han Chinese children	506	Risk of NR (Clinical and Radiological Diagnosis)	Increased risk of NR *P* = 0.019, OR: 1.467, 95% CI: 1.070-2.011		Zhang et al. (41)
			rs2060793	14893764	5’ UTR	Han Chinese children	506	Risk of NR (Clinical and Radiological Diagnosis)	Increased risk of NR rs2060793 G, *P* = 0.023, OR: 0.689, 95% CI: 0.502- 0.944		Zhang et al. (41)

### Vitamin D receptor

The *VDR* gene spans 63.49 kb on Chr12q13.11 (25). Missense and nonsense mutations in *VDR* cause hereditary vitamin D–resistant rickets (HVDRR), a syndrome of severe rickets that appears soon after birth (47). HDVRR and loss of function *VDR* mutations are covered in more detail elsewhere, however this condition demonstrates the importance of the VDR on normal bone metabolism.

As demonstrated in **Table 2**, there are several *VDR* polymorphisms implicated in nutritional rickets, with the most studied polymorphisms occurring in intron 8 (*Bsm* I (b alle) and *Apa* I sites), exon 9 (*Taq* I site), the 3′untranslated region (3’UTR), and exon 2 (*Fok1* site) (48).

The *VDR Fok1* restriction site defines a SNP in the first of two potential translation initiation start sites for the *VDR* gene. Two protein variants can exist corresponding to the two available start sites: the VDR encoded by the alternate allele form (ATG) (designated f), is three amino acids longer (48, 49). This has functional consequences for its intra-cellular activity as the amino acid structure created by the *Fok1*-f allele results in a less transcriptionally active VDR compared to the common allele form (ACG) (designated F). Several studies have associated VDR polymorphisms, especially *Fok1*, with the development of nutritional rickets. A study of 105 Nigerian children with nutritional rickets demonstrated that the *f* allele as less common in the rachitic group compared to the control group (0.17 vs 0.26, P = 0.03), with consistent results observed in Turkish (0.75 vs 0.65, *P = 0.024)* Chinese (0.37 vs 0.54, P <0.01) and Egyptian (0.25 vs 0.35, P = 0.024) cohorts (41–45). Additionally, El Kholy et al. found that several variant *VDR* SNPs including *Fok1*, were associated with phenotypic differences associated with nutritional rickets, including, baseline serum 25-OH-D and calcium, the X-ray severity score on diagnosis, time to radiologic healing and time to ALP normalisation (44). The *Fok1* f allele was also associated with a lower x-ray score in the rachitic children.

It is unknown exactly why the *ff Fok1 VDR* variant, which Is less transcriptionally active, is less common in patients with nutritional rickets. Additionally, there is controversy over whether the presence of this variant affects serum 25(OH)D or 1,25(OH)_2_ D levels. A systematic review of *VDR* polymorphisms demonstrated that in total, *VDR* polymorphisms were associated with a change in 25(OH)D of 1,25(OH)_2_D in 17 % of the included studies (50). However, a separate meta-analysis identified that the more biologically active *FF* genotype of the *Fok1* polymorphism was associated with an improved response to vitamin D supplementation, suggesting that the activity of the VDR might be associated with the response to vitamin D supplementation (51). Although these studies suggest that VDR polymorphisms are implicated in nutritional rickets, it appears that different modifying or environmental factors can interact with these genotypes, which may explain the different behaviours in different populations.

### Vitamin D binding protein (GC)

*GC* (group-specific component) encodes the vitamin D binding protein (VDBP) and is located on chromosome 4, covering 63.84 kb (52). At least 13 SNPs in the *GC* gene have been associated with circulating 25(OH)D levels, however the two most studied SNPs are in exon 11: rs7041 (G/T) and rs4588 (C/A) that causes a Glu/Asp amino acid and Thr/Lys amino acid change at codon 416 and 420, respectively. Together, they define the GC1s (TC), GC1f (GC), and GC2 (TA) haplotypes (53).

Rs4588 (C) and Rs7041 (T) carriers have significantly lower serum 25(OH)D levels in Caucasian, African American, and Han Chinese populations (32, 53–56). Carriers of one or more GC2 alleles were also found to have significantly lower serum concentrations of 25(OH)D with GC2/GC2 homozygotes more likely to be categorised as deficient (57, 58). Several polymorphisms in *GC* were associated with an increased frequency of nutritional rickets in one study of Han Chinese children (**Table 2**), including rs4588 (41). However, these results were not replicated in studies of other populations.

## Genome wide association studies

Genome wide association studies (GWAS) involve testing genetic variants across the genomes of unrelated individuals to identify genotype-phenotype associations (57). This approach allows for associations between SNPs and various phenotypes, biological pathways and diseases to be investigated. However, only common SNPs (with a minor allele frequency typically >1%) are usually targeted by GWAS. Although there are no GWAS that have specifically looked at nutritional rickets in humans, several have been performed on genetic factors affecting serum vitamin D. The first GWAS of vitamin D was conducted in 2007 among 517 related individuals of European ancestry but failed to identify any SNPs that were associated with 25(OH)D levels at genome-wide significance (59). However, this study was underpowered, with GWAS often requiring very large sample sizes to detect disease loci with small effect sizes (60). Therefore, meta-analyses of multiple GWAS have been used to identify common SNPs of low effect size. Key findings were initially reported in two meta-GWAS – one of participants of European ancestry that studied 6722 participants, and the SUNLIGHT consortium, which studied 33,996 participants (61, 62). The results from these two meta-GWAS showed a strong genome wide association with serum 25(OH)D at three loci at *GC* (index SNP: rs2282679), *DHCR7*/*NADSYN1* (rs12785878) and *CYP2R1* (rs10741657) as well as one novel locus at *CYP24A1* (rs17216707) (61, 62). An expanded SUNLIGHT meta -GWAS (that increased the sample size to 79,366) identified two novel loci with variants at *SEC23A* and *AMDHD1*, which are genes with a primary function outside of the vitamin D metabolism pathway (63). This work has been expanded upon by a subsequent GWAS of UK Biobank data, which identified 143 loci associated with 25(OH)D concentration in 417,580 Europeans (64). This included confirmation of the previously identified SNPs, as well as loci associated with skin colour that were not related to pigmentation such as dermal integrity (*FLG*), development (*PADI*), and UVB absorption in the skin (*HAL*). GWAS have also been conducted in populations of different ethnicities, used to determine both trans-ethnic as well as ethnicity-specific loci of interest. The Trans-Ethnic Evaluation of vitamin D (TRANSCEN-D) study compared data from the SUNLIGHT meta-GWAS to data from participants of Hispanic and African ancestries, replicating the association between SNPs in *GC* and *DHCR7* and 25(OH)D level as well as identifying novel SNPs near *KIF4B, ANO6/ARID2*, and *HTR2A* (65). A GWAS of 12,642 Korean participants identified a novel polymorphism in *ACTE1P*, which is 0.01Mb upstream of the *DHCR7* locus and may potentially be related to its gene function (66). Additionally, this has given insights into the factors that may influence the response to vitamin D supplementation – one GWAS of 761 Finnish children showed SNPs on *GC* and *CYP2R1* were associated with a decreased response to vitamin D supplementation (67).

GWAS have allowed us to develop our current understanding of the genetic contributions to nutritional rickets by providing potential SNPs that may be associated with 25(OH)D status, which may then provide insight into the genetic contribution to nutritional rickets based on further mechanistic evaluations. However, there are several limitations in using GWAS to characterise nutritional rickets. While GWAS have so far identified multiple independent loci associated with vitamin D levels, known SNPs still account for less than 20% of the total variance in heritability, leaving a large proportion of that demonstrated in twin/family studies unexplained (60, 63). This suggests that most of the genetic determinants affecting the formation of nutritional rickets and vitamin D status may be accounted for by rare SNPs for which current sample sizes and GWAS methodology are underpowered to detect.

Like most other genetic techniques that have been discussed, current GWAS has focused on vitamin D status. Although vitamin D and calcium status is important in the development of nutritional rickets, it can only serve as an indicator for the potential risk of developing nutritional rickets based on our prior understanding the role of vitamin D metabolism in its development. This also means that we cannot associate genotypes with the frequency of developing nutritional rickets or disease severity. However, by highlighting variants of interest, follow up work could look at the relationship between disease specific data (such as biochemical or radiological findings in rickets) and SNPs generated by GWAS on 25(OH)D.

## Epigenetic studies

### Histone modification and DNA methylation

Understanding the role of gene-environmental interactions is important in increasing our understanding of nutritional rickets by determining the ‘population-attributable’ effects of environmental exposures (such as UVB exposure) on the biological pathways implicated in nutritional rickets. Epigenetic mechanisms refer to modifications to gene expression by both the environment and behavioural factors and can explain how individual gene variants may not be expressed equally in all individuals (68). Although there is a lack of evidence associating epigenetic factors with radiologically/biochemically confirmed nutritional rickets, environmental factors have been reported to have an effect on genes implicated in the development of nutritional rickets. A systematic review of 7 studies comprising of 332,418 participants demonstrated that the genotypic effect of several gene polymorphisms were modulated, at least in part, by sun exposure (69). This includes the *Fok1 VDR* polymorphism, as well as polymorphisms in *GC*, *SEC23A*, *CYP2R1*, and *CYP27A1*. However, the authors noted several limitations, including heterogeneity in study designs (including differing climates and measures for sunlight exposure), as well as the relatively small sample size and the lack of availability of non-European populations (69).

One key mechanism of epigenetic modification includes methylation of DNA and covalent modifications of histones by methylation, acetylation, phosphorylation, or ubiquitination. Methylation is mediated by nuclear enzymes, such as DNA methyltransferases (DNMTs) and ten-eleven translocation (TET) proteins, while enzymes such as histone acetyltransferases (HATs), histone deacetylases (HDACs), histone methyltransferases (HMTs), and histone demethylases (HDMs) regulate histone modifications (68).

Methylation around 2 kb upstream from the promoter region of the *RXRA* gene (that encodes RXR) in the umbilical cord was associated with a lower offspring bone mineral content in two independent cohort studies and *RXRA* methylation at one CpG site was inversely related to serum 25(OH)D concentration (70–72).

Vitamin D is an epigenetically active molecule, with methylation of genes related to vitamin D metabolism shown to be associated with vitamin D status. For example, 25(OH)D deficient subjects showed increased methylation in CYP2R1 and a decreased methylation of CYP24A1 compared to vitamin D sufficient subjects (73). Further work demonstrated a negative relationship between CYP2R1 and CYP27B1 methylation and circulating 25(OH)D levels. These findings suggest that the methylation of these genes may contribute to vitamin D deficiency *via* reduced conversion of 25(OH)D to 1,25(OH)_2_D and increased conversion to inactive metabolites.

Methylation status is also associated with subject response to vitamin D supplementation. Post-menopausal women who were non responders to vitamin D supplementation had a higher methylation status of *CYP2R1* at baseline and after supplementation (74). Although vitamin D supplementation had no effect on *CYP27B1* in this study, supplementation was associated with a decrease in the methylation levels of *CYP24A1*. Additionally, interventional studies observing the effect of both long-term vitamin D supplementation (the VitDmet study) and a single vitamin D bolus (the VitDBol study) demonstrated that higher mRNA expression changes of vitamin D target genes were correlated to the change in vitamin D status (75). These were used to provide transcriptomic biomarkers that were used to create a ‘vitamin D response index’ that could be used to segregate participant’s responses (76). This indicated that around 25% of the study participants were low responders to vitamin D supplementation (75, 76). The clinical relevance of this was theoretically applied to the United Arab Emirates, where 82.5% of hospitalised patients showed vitamin D insufficiency (77). They then extrapolated that 25% of this population may be low responders and speculated that screening patients using a vitamin D bolus (with a similar method to the VitDbol study) might determine a patient’s vitamin D response index, which could influence treatment regimens (76). This data suggests that the epigenome is important in regulating some of the pathways that have been implicated in the development of nutritional rickets. However, the application of how this might influence treatment is limited to speculation at present, as the percentage that the epigenome that contributes to the risk of developing nutritional rickets remains unknown.

Despite the information given by such studies, analyses of current assays are limited as only a tiny percentage of the number of potentially methylated CpG sites across the genome have been targeted; and although the variance explained by molecular phenotype is generally greater than that explained by simple genetic variation, the sample sizes of these studies have been relatively small compared with what has recently been possible. Future work involving collaborative efforts to investigate methylation sites across the whole genome will provide greater insights. Furthermore, DNA methylation and histone modifications represent only a subset of mechanisms for epigenetic modification of gene expression. Other factors such as chromatin accessibility at different times in the life course, and an expanding range of non-coding RNAs also affect gene transcription and translation to influence gene expression.

### Noncoding RNAs

Advances in transcriptomic analysis through techniques such as RNA-seq have allowed the identification of novel and diverse non-coding RNAs (ncRNAs). Nc-RNAs are clusters of RNAs that are not translated into functional proteins and can be divided into two categories based on size – short chain non-coding RNAs (including miRNAs, piRNAs, siRNAs and cirRNAs) and long non-coding RNAs (lncRNAs) (78). Evidence accumulated over the last 10-15 years suggests that nc RNAs play a significant role in epigenetic modification, occurring at the level of the gene and/or at the level of the chromosome (79).

MicroRNAs (miRNAs) are small (19–24 nucleotides long) non-coding RNA molecules that participate in the post transcriptional modulation of gene expression by binding to the 3’-UTR of mRNA transcripts (80). This results in reduced protein synthesis by directly repressing translation and/or accelerating mRNA degradation. Although miRNAs account for only 1-5% of the human genome, miRNAs have been implicated in many different processes including the development of cardiovascular diseases, COVID-19, diabetes, and cancer through controlling the translation of mRNA or through affecting distant tissue responses (81–83). Because of this, miRNAs have been highlighted for their potential to serve as both biomarkers and therapeutic targets of disease (84, 85). MiRNAs also regulate vitamin D metabolism, and currently identified targets include CYP27B1, CYP24A1 and VDR (81, 86). This information was initially based on in-silico models; however, several targets have been experimentally validated (87). MiR-21 has been shown to suppress expression of CYP27B1, while miR-125b has been shown to target the VDR and CYP24A1 mRNAs (86–88). Additionally, vitamin D metabolites have been shown to impact the expression of certain miRNAs. Peripheral blood concentrations of 11 miRNAs were associated with circulating 25(OH)D levels in pregnancy (89). In another study, the expression of miR-21 was also higher in the aorta and internal mammary arteries of 25(OH)D deficient patients who underwent coronary artery bypass grafting (90). However, it was subsequently shown that correcting 25(OH)D levels did not affect circulating miR-21 levels despite improving different markers of cardiovascular health (91).

In the context of bone metabolism, miRNA dysregulation has also been linked with the development of several bone diseases, such as osteoporosis, osteoarthritis as well as primary and secondary bone malignancies (81). For example, human osteoblast cultures treated with 1,25(OH)_2_D showed differential expression of miR-1228 and miR-637 (92). Additionally, miR-21 has been demonstrated to promote osteogenic differentiation in human BMSCs by increasing the mRNA expression of several osteogenic markers including *Runx2*, *osteonectin* and *SOX2* (93, 94). It has demonstrated in mouse osteoblasts that upregulation of *Runx2* by miRNAs is at least partially achieved by decreasing the activity of histone deacetylase 5 (HDAC5) which acts a transcriptional repressor of *Runx2*, causing a subsequent increase in Runx2 transcriptional activity (95, 96).

Long noncoding RNAs (lncRNAs) are a family of non-coding RNA transcripts that are normally 200 -100,000 nucleotides long and make up approximately 80% of the transcriptome originating from a multitude of different regions on genes (97). Although lncRNAs share structural similarities to mRNA, they demonstrate a substantially lower protein coding potential and many of the 28,000 known lncRNAs are poorly understood (98). However, lncRNAs play key roles in regulating gene expression in growth and development. LncRNA activity has also been implicated in bone homoeostasis, primarily in osteoblast and osteoclast differentiation (79). For example, human BMSC knockouts of the LncRNA *DANCR* – Differentiation Antagonizing Non-Protein Coding RNA demonstrated enhanced mRNA expression of osteogenic marker genes and mineralised matrix deposition. This has been shown to occur *via* several processes, including the inhibition of markers such as *Runx2* and increased *TNFα* expression (79, 99). In addition to the direct effect of lncRNAs, lncRNAs have been seen to interact with miRNAs in the regulation of the proliferation of osteoblasts and osteoclasts. For example, lncMALAT1 and lncTUG1 positively regulate *Runx2* expression by ‘sponging’ antidifferentiation miRNAs, acting to decrease their effective concentration and activity and therefore promoting osteoblast differentiation (100).

Although evidence suggests that non-coding RNAs are important in bone metabolism, relatively little is known regarding how ncRNAs specifically may be involved in the development of nutritional rickets. However, based on the above findings applying the current research into osteoblast differentiation and development to future research focusing on the effect of ncRNAs on the processes underlying endochondral ossification may be fruitful. For example, *in vitro* studies may look at the ncRNA transcription profiles of chondrocyte constructs in response to differing concentrations of 1,25(OH)_2_D and the relationship with expression of various proteins involved in extracellular matrix formation and chondrocyte apoptosis. This could be supplemented by in studies analysing the transcription profile of various ncRNAs of interest in patients with nutritional rickets to establish any potential relationships, which may yield both diagnostic and therapeutic benefits. Future research exploring epigenetic mechanisms will be critical in identifying functionally relevant loci that contribute to the environmental impact (whether *via* sunlight exposure or maternal and childhood dietary indices). Additionally, once areas of interest have been identified, future work to identify whether these variations are associated with both –cis and –trans gene-gene interactions would further help resolve the epigenetic and genetic determinants of nutritional rickets.

## Conclusion

In this review, we briefly summarise a complex interplay between genetic and epigenetic factors that might influence an individual’s 25(OH)D status and risk of developing nutritional rickets. However, due to the lack of direct studies on nutritional rickets, there remains very little information on whether the factors mentioned might affect the development of the phenotypical features of rickets in vitamin deplete children and the reasons for why some children can be substantially vitamin D/calcium deficient and not develop symptoms. Future studies could build on the work described in this review by focusing specifically on phenotypic manifestations of nutritional rickets (rather than a generic focus on vitamin D) and if variations in already described genes lead to a clinical difference. This would help to develop our knowledge of nutritional rickets and hopefully aid diagnosis and management in the future.

## Author contributions

IO, AA and JW contributed to the design and implementation of the review and the formation of the manuscript. All authors contributed to the article and approved the submitted version.
